# British HIV and ageing study. HIV and ageing: older people with HIV, who are they?

**DOI:** 10.1186/1758-2652-13-S4-P57

**Published:** 2010-11-08

**Authors:** S Allan, RA Daly, Y Yoganathan, S Barrett, A Joseph, A Tariq, CW Saing, C Williams, C Lane, J Sikorska

**Affiliations:** 1 Coventry and Warwickshire Hospital, GU/HIV Medicine, Coventry, UK; 2 Singleton Hospital, Sketty Lane, Swansea, UK; 3 Heartlands Hospital, GU/HIV Medicine, Birmingham, UK; 4 New Cross Hospital, GU/HIV Medicine, Wolverhampton, UK; 5 Manor Hospital, GU/HIV Medicine, Walsall, UK

## Objectives

The life expectancy of HIV positive individuals has improved dramatically with the advent of HAART. The cohort of older people living with HIV is steadily increasing in size. Both advancing age and HIV are associated with an increased likelihood of comorbidities and polypharmacy independently, but little is known about their combined impact and how this will shape the care required from HIV physicians. This study was designed to gain insight into the characteristics of patients aged 60 or over living with HIV.

## Methods

Data was collected from 5 centres across England and Wales. All patients currently aged 60 or over were included. A questionnaire format was used to collect information on demographics, risk factors for infection, co-morbidities, co-infections, all medicine use including HAART and resistance patterns.

## Results

There were 66 patients in total. 79% of these patients were male. Half of the patients were MSM. 76% were Caucasian, 12% Black African, 8% Black Caribbean and 3% Asian. The current age range was 60 to 79 years, with a mode and median age of 62 and 64 years respectively. The age of diagnosis ranged from 39 to 71, with a median age of 59 years. Almost half of the patients were diagnosed at age 60 or over. The duration of HIV infection ranged from 2 months to 22 years with 48% of patients having been diagnosed within the last 5 years. This supports a combination of patients living to older ages with HIV and acquisition of HIV in later life. 53% had a CD4 count of less than 200 at diagnosis. 38% had had an AIDS defining condition. Only 7% had a CD4 count of <200cells/mm^3^ in 2010. 84% had one or more comorbidity. 31% had 3 or more comorbidities. 92% of patients were on HAART. Of those on treatment, 86% had current viral loads of <50 copies/ml. Of the 5 patients who were not on treatment, all were between the age of 60 and 65 currently, diagnosed within the last 4 years and had CD4 counts over 450cells/mm^3^. 89% of patients were on 1 or more prescribed medication, in addition to HAART or septrin. 23% were on 5 or more medications.

## Conclusions

This data provides an insight into an ever growing cohort of older people with HIV, in whom there is a paucity of information. It gives some information on the risk factors, comorbidities, stage of infection at diagnosis and response to treatment. In this cohort, despite the complexities associated with managing HIV in older patients, the majority of patients were fully suppressed with good CD4 counts.

